# Adiposity markers and risk of coronary heart disease in patients with type 2 diabetes mellitus

**DOI:** 10.1186/1475-2891-13-124

**Published:** 2014-12-23

**Authors:** Simone F Tonding, Flávia M Silva, Juliana P Antonio, Mirela J Azevedo, Luis Henrique S Canani, Jussara C Almeida

**Affiliations:** Endocrinology Division, Hospital de Clinicas de Porto Alegre, Rua Ramiro Barcelos 2350, Prédio 12, 4° andar, 90035-003 Porto Alegre, RS Brazil; Graduate Program in Medical Sciences: Endocrinology, Universidade Federal do Rio Grande do Sul, Porto Alegre, Brazil; Department of Internal Medicine, Faculdade de Medicina, Universidade Federal do Rio Grande do Sul, Porto Alegre, Brazil

**Keywords:** Anthropometry, Body fat distribution, Obesity, Abdominal, Coronary disease, Diabetes mellitus, Type 2

## Abstract

**Background:**

This cross-sectional study aimed to evaluating the association between body adiposity markers and high-risk of coronary heart disease (CHD) in patients with type 2 diabetes.

**Methods:**

Recent adiposity markers [waist-to-height ratio, conicity index (C-index) and body adiposity index] and traditional markers [BMI, waist circumference and waist-to-hip ratio (WHR)] were measured. The 10-year risk of fatal CHD was estimated according to *UKPDS* risk engine scores. Patients were divided into high (CHD risk ≥20%; n = 99) or low-moderate (CHD risk <20%; n = 321) risk groups. Multiple logistic regression models were performed to analyze associations between CHD risk (outcome) and adiposity markers.

**Results:**

A total of 420 patients with type 2 diabetes (61.9 ± 9.5 years; 53.5% females; HbA1c 7.6 ± 1.6%) were evaluated. The high risk group had greater proportions of elevated C-index and BMI values than patients with low-moderate risk. No between-group differences in other adiposity markers were observed. In multiple logistic regression models, only C-index values ≥1.35 were associated with CHD risk >20% (OR = 1.69; 95% CI 1.03-2.78; P = 0.039) after adjusting for confounders (sedentary lifestyle, diabetic nephropathy, serum creatinine, and diabetes duration). The association between WHR and CHD risk did not hold in this sample.

**Conclusions:**

The C-index was the body adiposity marker best associated with high risk of fatal CHD in these patients with type 2 diabetes.

## Background

Cardiovascular disease is the leading cause of morbidity and mortality in patients with type 2 diabetes [1, 2]. In these patients, the risk of death from vascular causes is 2.32 times higher than in persons without diabetes [3], and diabetes was once considered a coronary heart disease (CHD) risk equivalent [2]. This concept was reinforced by a seminal population-based cohort study conducted in Finland in the late 1990s [4], which study suggested that the risk of coronary events of diabetic patients without previous myocardial infarction was similar to that of nondiabetic patients with a history of myocardial infarction. However, this observation was not confirmed in other population samples [5]. Indeed, it is now recognized that CVD risk varies among patients with diabetes, and that accurate estimation of risk clearly depends on individual characteristics [6]. Hence, the identification of patients with elevated cardiovascular disease risk plays an important role in the development of strategies to prevent cardiovascular events and to reinforce existing interventions. The American Diabetes Association has recommended individualized risk assessment for primary prevention using designed risk prediction models and algorithms [6].

As obesity is present in 80% of patients with type 2 diabetes [1] and is considered an independent risk factor for cardiovascular disease, its evaluation and treatment in these patients is extremely important [7]. Anthropometric markers are useful tools for assessment of overweight and/or obesity in clinical practice, and it has been suggested that these markers may be good predictors of cardiovascular risk [8]. BMI is the main predictor used to quantify body mass related to height, whereas waist circumference (WC), waist-hip ratio (WHR), waist-height ratio (WHtR), and the conicity index (C-index) are markers of central body fat accumulation, and the body adiposity index (BAI) is a predictor of whole body adiposity [9].

The WHR and WHtR are good predictors of cardiovascular risk in patients with type 2 diabetes [10, 11] and are considered traditional anthropometric markers, as are the BMI and WC. The role of these measurements in CHD risk assessment has been recently debated [12], and WHtR seems to be a more accurate risk marker than BMI and WC [13, 14] in the general population and in a sample of Chinese patients with diabetes [11]. Some novel body adiposity markers (C-index and BAI) have also been proposed, but evidence on their association with cardiovascular risk in patients with diabetes is scarce. This study sought to evaluate the potential association between body adiposity markers and high-risk of CHD as determined by the United Kingdom Prospective Diabetes Study (UKPDS) score in outpatients with type 2 diabetes.

## Methods

### Study population

This cross-sectional study was conducted in patients with type 2 diabetes from the Group of Nutrition in Endocrinology cohort. The study sample comprised 420 patients with type 2 diabetes (53.5% women, mean age 61.9 ± 9.5 years). All patients underwent clinical, anthropometric, and laboratory assessment. The diagnosis of diabetes was established if the fasting plasma glucose was ≥ 126 mg/dl or the 2-h plasma glucose value after a 75-g oral glucose tolerance test was ≥200 mg/dl. In the absence of unequivocal hyperglycemia, these results were always confirmed. Type 2 diabetes was defined as onset of hyperglycemia after the age of 30 years, with no history of ketoacidosis or documented ketonuria, and no insulin treatment in the first 5 years after diagnosis of diabetes [1]. Patients seen consecutively by the nutrition team at the Endocrinology Division of Hospital de Clinicas de Porto Alegre, Brazil, recruited from 2001 to 2010, were selected on the basis of the following criteria: age <80 years; serum creatinine <2.0 mg/dl; normal liver and thyroid function tests; absence of urinary tract infection or other renal disease; and absence of severe autonomic neuropathy (presence of symptomatic postural hypotension, gastroparesis or diabetic diarrhea). The study was conducted in accordance with Declaration of Helsinki guidelines, and all procedures involving patients were approved by the Hospital de Clinicas de Porto Alegre Research Ethics Committee. Written informed consent was obtained from all patients.

### Clinical and anthropometric evaluation

Sitting blood pressure was measured twice, after a 10-minute rest, using a digital sphygmomanometer (Omron HEM-705CP Kunotsubo, Terado-cho, Muko, Kyoto, 617–0002, Japan). Hypertension was defined as blood pressure ≥140/90 mmHg on two separate occasions or use of antihypertensive drugs [2]. According to a random spot urine sample or 24-h timed urine collection, patients were defined as normoalbuminuric (urinary albumin excretion [UAE] <17 mg/l or <20 mcg/min), microalbuminuric (UAE 17–174 mg/l or 20–199 mcg/min), or macroalbuminuric (UAE >175 mg/l or >199 mcg/min). The diagnosis of micro- and macroalbuminuria was always confirmed in two out of three urine samples [15]. A dilated fundus examination was performed and diabetic retinopathy was graded when present [16]. Physical activity was graded in levels according to activities during a typical day, based on a standardized questionnaire [17] adapted to local habits (namely, skiing was removed from the physical activity options). Four levels were defined, ranging from physically inactive to high physical activity. Patients were considered physically inactive when their activities during a typical day were best represented by the sentence "I read, watch television and do housework with little physical effort". Alcohol intake was considered positive in patients who mentioned current intake of any alcoholic beverage in a short standardized questionnaire. Patients were classified as current smokers or non-smokers. Ethnicity was self-reported as white (Latino) or non-white.

Body weight and height (measured with patients barefoot and wearing light clothing) were obtained using an anthropometric scale (Filizola, Filizola Balanças Industriais S.A., São Paulo, Brazil), with measurements recorded to the nearest 50 g for weight and to the nearest 0.1 cm for height. WC was measured at the midpoint between the lowest rib and the iliac crest, and hip circumference, at the most prominent point of the gluteus maximus [18]. Both measurements were obtained using flexible, non-stretch fiberglass tape. Based on anthropometric and laboratory data, the body adiposity markers of interest were estimated using the formulas described below:

1. **Body Mass Index (BMI)** [19]**:** body weight (kg)/ height (meters) squared
2. **Waist-to-hip ratio (WHR)** [19]**:** waist circumference (cm)/ hip circumference (cm)
3. **Waist-to-height ratio (WHtR)** [20]**:** waist circumference (cm)/ height (cm)
4. **Conicity index (C-index)** [21]**:**
5. **Body Adiposity Index (BAI)** [9]**:**

No specific model for prediction of cardiovascular risk in Latin American populations exists. Therefore, the risk of a CHD event was estimated using the UKPDS risk engine score [22]. This score has been validated specifically for patients with diabetes [23] and provides an estimate of risk for a new CHD event and stroke (fatal and nonfatal) at 5 and 10 years [22].

The data used to calculate the UKPDS risk score (available at http://www.dtu.ox.ac.uk/riskengine/download.php) were: glycosylated hemoglobin (HbA1c), systolic blood pressure, serum total cholesterol and high-density lipoprotein (HDL) cholesterol, atrial fibrillation, gender, age, ethnicity, smoking, and diabetes duration. Fatal CHD risk was estimated for 10 years [22]. Subjects with risk scores higher than 20% were classified as high-risk, whereas those with a risk score lower than 20% were classified into the low-moderate risk group.

### Laboratory measurements

Blood samples were obtained after a 12-h fast. Plasma glucose was determined by a glucose oxidase method [24], hemoglobin A_1c_ (reference range, 4.7 to 6.0%) by HPLC (Tosoh 2.2 Plus HbA_1c_; Tosoh Corporation, Tokyo, Japan) [25], total cholesterol [26] and triglycerides [27] by enzymatic colorimetric methods (Merck Diagnostica, Darmstadt, Germany; Boehringer Mannheim, Buenos Aires, Argentina), and high-density lipoprotein cholesterol (HDL) by the homogeneous direct method (enzymatic colorimetric reaction, ad described by Fletcher and modified by Farish) [28]. Low-density lipoprotein cholesterol (LDL) was calculated using Friedewald’s formula [29] only for patients with values <40 0 mg/dl. UAE was measured by immunoturbidimetry (MicroAlb Sera-Pak® Immunomicroalbuminuria; Bayer, Tarrytown, NY, USA) on a Cobas Mira Plus® analyzer (Roche, Indianapolis, IN, USA) [30]. Creatinine values were quantitated by Jaffe’s reaction [31].

### Statistical analyses

Considering that this study aimed to evaluate the association between body adiposity markers and high risk of CHD, patients were divided into high-risk (CHD risk ≥20%; n = 99) or low-moderate risk (CHD risk <20%; n = 321) groups, and their demographic, clinical, and laboratory parameters were compared by Student’s *t*-test, the Mann–Whitney *U* test, and the chi-square test as appropriate. All analyses were carried out in the PASW Statistics 18.0 software suite (SPSS Inc., Chicago, IL). Data are described as mean ± SD, median (interquartile range), or n (%). The type I error rate was set at P < 0.05 (two-tailed).

The adiposity markers were converted to categorical variables (normal *vs.* abnormal values) in accordance with established cutoff values available in the literature (BMI ≥30 kg/m^2^ [19], WC >80 cm for females or >94 cm for males [32], WHR >0.85 for females or >0.90 for males [16], and WHtR >0.5 [33]). Regarding the BAI and C-index, mean values – for the C-index, > 1.35; for the BAI, >35 for females or >25 for males – were adopted due to absence of established cutoff values. The receiver operating characteristic (ROC) curve performance of these adiposity markers to identify 10-year CHD risk in type 2 diabetes has been demonstrated elsewhere, and the C-index and BAI cutoff values were identified by sensitivity and specificity equilibrium [34]. The association between adiposity markers (dichotomous variable) and presence of high CHD risk (dependent variable) was evaluated by multiple logistic regression analyses, adjusted for potential confounders selected according to clinical relevance or significance on univariate analyses.

## Results

The study sample comprised 420 patients with type 2 diabetes, of whom 23.6% were classified as being at high risk (UKPDS risk score >20%) for fatal CHD events; mean UKPDS score was 37.0 ± 11.4% (95% CI 34.7 – 39.3%). The demographic, clinical, lifestyle, and laboratory characteristics of patients according to CHD risk are shown in Table 1. Patients in the high-risk group had a higher proportion of micro- and macroalbuminuria, were more physically inactive, and had higher fasting plasma glucose, LDL-cholesterol, triglycerides, and serum creatinine levels than did patients in the low-moderate risk group (all *P* values < 0.05). The parameters that represent the components of the UKPDS risk score were, as expected, significantly different between the two groups, except for HDL-cholesterol values in women (*P* = 0.246). Evaluation of diabetic retinopathy was available for 307 subjects. In this subgroup, patients with a high CHD risk had a higher proportion of diabetic retinopathy (53.3%) than patients with low-moderate risk (38.4%; *P* = 0.031).

**Table 1:** **Demographic, clinical, and laboratory profile of patients with type 2 diabetes, stratified by CHD risk**

	All patients	High risk (≥20%)	Low-moderate risk (<20%)	P-value
N	420	99	321	**-**
Female	226 (53.5%)	31 (31.3%)	195 (60.7%)	**-**
Age (years)	61.9 ± 9.5	68.1 ± 6.4	58.7 ± 9.2	**-**
Diabetes duration (years)	10.0 (6.0-17.0)	15.0 (9.0-22.0)	10.0 (5.0-15.0)	**-**
White (Latino) ethnicity	348 (82.9%)	94 (94.9%)	254 (79.1%)	**-**
Education (years)	7.2 ± 3.6	7.0 ± 3.5	7.3 ± 3.6	0.554^a^
Smoking				
Current smokers	48 (11.4%)	13 (13.1%)	35 (10.9%)	
Former smokers	175 (41.7%)	52 (52.5%)	123 (38.3%)	-
Nonsmokers	197 (46.9%)	34 (34.3%)	163 (50.8%)	
Current alcohol intake	132 (31.4%)	33 (33.3%)	99 (30.8%)	0.186^b^
Sedentary lifestyle	246 (58.7%)	63 (63.6%)	183 (57.2%)	*<0.001* ^*b*^
Micro- and macroalbuminuria	129 (31.0%)	41 (41.8%)	87 (27.4%)	0.007^b^
Current use of hypolipidemic drugs	151 (36.0%)	36 (36.4%)	115 (35.8%)	0.922^b^
Diabetes treatment				
Diet	24 (5.7%)	3 (3.0%)	21 (6.5%)	
Oral antidiabetics	245 (58.3%)	50 (50.5%)	195 (60.8%)	0.082^b^
Oral antidiabetics and/or insulin	151 (36.0%)	46 (46.5%)	105 (32.7%)	
Hypertension	360 (85.7%)	85 (85.9%)	257 (81.0%)	0.195^b^
Systolic blood pressure (mmHg)	139 ± 21	143 ± 24	137 ± 20	-
Diastolic blood pressure (mmHg)	80 ± 12	79 ± 14	80 ± 12	0.407^a^
BMI (kg/m^2^)	28.8 ± 4.3	28.2 ± 4.5	28.9 ± 4.3	0.163^a^
Fasting plasma glucose (mg/dl)	149.8 ± 55.6	166.3 ± 64.8	144.7 ± 51.4	0.003^a^
Glycosylated hemoglobin (%)	7.6 ± 1.6	8.3 ± 2.0	7.3 ± 1.4	-
Total cholesterol (mg/dl)	201.2 ± 41.6	212.5 ± 42.5	197.7 ± 40.7	-
HDL cholesterol (mg/dl)				
Female (n = 226)	51.9 ± 12.8	49.4 ± 11.5	52.3 ± 13.0	*-*
Male (n = 194)	46.2 ± 11.3	43.5 ± 10.0	47.7 ± 11.8	*-*
LDL cholesterol (mg/dl)	121.0 ± 35.5	130.6 ± 39.3	116.9 ± 34.4	0.002^a^
Triglycerides (mg/dl)	135.5 (100.0-201.0)	159.0 (117.0-236.0)	132.0 (94.5-186.0)	*<0.001* ^*c*^
Serum creatinine (mg/dl)	0.87 ± 0.23	0.94 ± 0.26	0.84 ± 0.22	*<0.001* ^*a*^

Data expressed as means ± SD, median (interquartile range), or n (%). CHD, Coronary heart disease. Current alcohol intake available for 308 patients.

^a^Student t test, ^b^Chi-square test, ^c^Mann–Whitney U test.

We conducted analyses considering adiposity marker values as categorical variables. The proportion of patients with type 2 diabetes by CHD risk group with abnormal values of each adiposity marker is shown in Table 2. The majority of patients had abnormally high WC (86.0%), WHR (94.8%), or WHtR (96.9%), though we did not observe any difference between the high and low-moderate CHD risk groups. Conversely, greater proportions of patients (both male and female) in the high-risk group had elevated C-index and BMI values than in the low-risk group. Half of all patients had elevated BAI values (49.5%), with no significant between-group difference.

**Table 2:** **Adiposity marker classification in patients with type 2 diabetes, stratified by CHD risk**

	All patients	High risk (≥20%)	Low-moderate risk (<20%)	*P* ^*a*^
**n**	420	99	321	-
**Body mass index classification (WHO criteria)** ^**b**^	162 (38.6%)	30 (30.3%)	132 (41.1%)	*0.030*
**Obesity (≥30.0 kg/m** ^**2**^ **)**				
**Waist circumference (IDF criteria)** ^**c**^ **:**	361 (86.0%)	83 (83.8%)	278 (86.6%)	0.294
**>80 cm for females and >94 cm for males**				
**Waist-to-hip ratio (WHO criteria)** ^**b**^ **:**	398 (94.8%)	97 (98.0%)	301 (93.8%)	0.080
**>0.85 for females and >0.90 for males**				
**Waist-to-height ratio** ^**d**^ **: >0.5**	407 (96.9%)	96 (97.0%)	311 (96.9%)	0.630
**Conicity index (mean): >1.35**	211 (50.2%)	60 (60.6%)	151 (47.0%)	*0.012*
**Body adiposity index (mean):**	208 (49.5%)	47 (47.5%)	161 (50.2%)	0.363
**>35 for females and >25 for males**				

CHD, coronary heart disease. The 10-year risk of fatal CHD was estimated according to UKPDS risk engine scores. Data are expressed as number of patients with the analyzed characteristic (%). ^a^Chi-square; abnormal levels for each adiposity marker defined as values higher than the sample mean (conicity and body adiposity indexes) or using well-known cutoffs established in the literature: ^b^WHO criteria (1998); ^c^International Diabetes Federation criteria for the European population (2005); ^d^Browning et al. (2010), Nutr Res Rev.

Multiple logistic regression models were constructed and used to evaluate possible associations between adiposity markers and high 10-year CHD risk scores (Table 3). Only the C-index was associated with high CHD risk: C-index values ≥1.35 increased the odds of high CHD risk significantly (OR 1.69; 95% CI 1.03 – 2.78; *P* = 0.039) after adjusting for confounders (sedentary lifestyle, diabetic nephropathy, serum creatinine, and diabetes duration). When retinopathy was included as a confounding variable in a sub-sample of patients (n = 307), the C-index association with the presence of high CHD risk followed a similar pattern (OR 1.91; 95% CI 1.08 – 3.35; *P* = 0.025).

**Table 3:** **Multiple logistic regression models: high 10-year CHD risk (≥20% by UKPDS score) as dependent variable**

	Abnormal levels	Abnormal levels - adjusted model
**Body mass index**	1.59 (0.98-2.58)	1.43 (0.85-2.42)
**Waist circumference**	1.23 (0.66-2.30)	1.07 (0.54-2.12)
**Waist-to-hip ratio**	0.32 (0.07-1.42)	0.38 (0.08-1.79)
**Waist-to-height ratio**	0.96 (0.26-3.57)	0.96 (0.23-4.01)
**Conicity index**	1.72 (1.09-2.73)*	1.69 (1.03-2.78)*
**Body adiposity index**	1,12 (0.71-1.76)	1.06 (0.65-1.73)

Analyses performed on 420 type 2 diabetic patients. Markers defined as abnormal when: Body Mass Index ≥30 kg/m^2^; waist circumference >80 cm for females or >94 cm for males; waist-to-hip ratio >0.85 for females or >0.90 for males; waist-to-height ratio >0.5; conicity index >1.35; body adiposity index >35 for females or >25 for males. Adjusted model: sedentary lifestyle, nephropathy, serum creatinine, and diabetes duration (years) as confounders. Normal levels were used as reference for regression models. *P <0.05.

## Discussion

In this study, a novel marker of adiposity, the C-index, was associated with the 10-year risk of fatal CHD events in patients with type 2 diabetes. C-index values above 1.35 increased the odds of high CHD risk by 69%, after adjusting for sedentary lifestyle, diabetic nephropathy, serum creatinine, and diabetes duration (years).

A positive association between the C-index and cardiovascular risk factors has already been demonstrated by other authors [35, 36], although none of these studies were conducted in patients with diabetes, precluding comparison with our results. Patients with type 2 diabetes exhibit increased visceral and intermuscular adiposity - which is related to the presence of insulin resistance - as compared with the general population [37] and this aspect may be explain partially the "Obesity Paradox" observed in our results. Although it does not distinguish between visceral and subcutaneous abdominal fat, the C-index is a comprehensive marker of abdominal adiposity, because the WC is adjusted for weight and height, which allows direct comparisons of fat distribution between individuals and populations [21].

Traditionally, WC has been considered a risk factor for all-cause mortality in adults, including cardiovascular disease [38], though this was questioned in a recent meta-analysis of 58 cohorts [12]. In the current study, WC alone did not show any significant association with cardiovascular risk. Possibly, WC measurement may be relevant when combined with others variables, especially height and weight, in the first step for identification of CHD risk (hard outcomes) in clinical practice (screening), but this hypothesis should be tested. In a systematic review and meta-analysis performed by Ashwell et al. [13], the WHtR is described as a better screening tool than WC to discriminate diabetes, hypertension, dyslipidemia, the metabolic syndrome, and cardiovascular outcomes in adults. However, in our sample, no association was observed between waist-to-height ratio and 10-year risk of fatal CHD events.

Possible limitations of our study concern adoption of the cardiovascular risk score as a surrogate endpoint instead of CHD events or mortality and the absence of a validated cardiovascular score for Brazilians or Latin Americans with diabetes. Furthermore, the utility of the UKPDS score in patients with diabetes duration of more than 10 years is unclear, as the UKPDS cohort included only newly diagnosed patients [22]. However, the impact of diabetes duration on the performance of the UKPDS risk engine was recently evaluated, and a similar discrimination of the model for patients with diabetes duration >10 years or <10 years was demonstrated [23]. Accordingly, when analysis of our study sample was restricted to patients with a diabetes duration of >10 years (48.8% of patients), we observed the same pattern of association between the C-index and high CHD risk (data not shown). Another potential limitation is the cross-sectional design of our study, which precludes evaluation of the studied adiposity markers as actual risk factors for CHD. Moreover, the C-index cutoff value needs to be determined considering hard CHD endpoints. Still, our exclusion criteria may have influenced in the proportion of patients with higher cardiovascular risk and/or diabetic complications, in this way, the association between C-index and cardiovascular risk needs to be tested in other populations of patients with type 2 diabetes.

The novelty of the present study lies in its demonstration that the C-index can make an important contribution to the interpretation of anthropometric parameters commonly used in clinical practice, such as weight, height, and WC. Furthermore, it can be readily used as a non-laboratory tool for CHD risk screening in patients with diabetes.

## Conclusion

The C-index was associated with 10-year risk of fatal CHD events in patients with type 2 diabetes. Nevertheless, the potential role of the C-index as a predictor of high CHD risk in patients with diabetes should be confirmed in prospective studies using hard endpoints.

## Abbreviations

CHD: Coronary heart disease
BMI: Body mass index
WC: Waist circumference
WHR: Waist-hip ratio
WHtR: Waist-height ratio
C-index: Conicity index
BAI: Body adiposity index
UKPDS: United Kingdom Prospective Diabetes Study
HbA1c: Glycosylated hemoglobin
HDL: High-density lipoprotein
LDL: Low-density lipoprotein
UAE: Urinary albumin excretion.
